# Resilience in Long-Term Viral Infection: Genetic Determinants and Interactions

**DOI:** 10.3390/ijms222111379

**Published:** 2021-10-21

**Authors:** Candice Brinkmeyer-Langford, Katia Amstalden, Kranti Konganti, Andrew Hillhouse, Koedi Lawley, Aracely Perez-Gomez, Colin R. Young, C. Jane Welsh, David W. Threadgill

**Affiliations:** 1 Department of Veterinary Integrative Biosciences, Texas A&M University, College Station, TX 77843, USA; kamstalden@cvm.tamu.edu (K.A.); koedilawley@tamu.edu (K.L.); aapg96@tamu.edu (A.P.-G.); cyoung@cvm.tamu.edu (C.R.Y.); jwelsh@cvm.tamu.edu (C.J.W.); 2 Texas A&M Institute for Genome Sciences and Society, Texas A&M University, College Station, TX 77843, USA; konganti@tamu.edu (K.K.); hillhouse@tamu.edu (A.H.); dwthreadgill@tamu.edu (D.W.T.); 3 Department of Veterinary Pathobiology, Texas A&M University, College Station, TX 77843, USA; 4 Department of Molecular and Cellular Medicine, Texas A&M University, College Station, TX 77843, USA

**Keywords:** TMEV, resilience, collaborative cross, gene expression

## Abstract

Virus-induced neurological sequelae resulting from infection by Theiler’s murine encephalomyelitis virus (TMEV) are used for studying human conditions ranging from epileptic seizures to demyelinating disease. Mouse strains are typically considered susceptible or resistant to TMEV infection based on viral persistence and extreme phenotypes, such as demyelination. We have identified a broader spectrum of phenotypic outcomes by infecting strains of the genetically diverse Collaborative Cross (CC) mouse resource. We evaluated the chronic-infection gene expression profiles of hippocampi and thoracic spinal cords for 19 CC strains in relation to phenotypic severity and TMEV persistence. Strains were clustered based on similar phenotypic profiles and TMEV levels at 90 days post-infection, and we categorized distinct TMEV response profiles. The three most common profiles included “resistant” and “susceptible,” as before, as well as a “resilient” TMEV response group which experienced both TMEV persistence and mild neurological phenotypes even at 90 days post-infection. Each profile had a distinct gene expression signature, allowing the identification of pathways and networks specific to each TMEV response group. CC founder haplotypes for genes involved in these pathways/networks revealed candidate response-specific alleles. These alleles demonstrated pleiotropy and epigenetic (miRNA) regulation in long-term TMEV infection, with particular relevance for resilient mouse strains.

## 1. Introduction

Theiler’s murine encephalomyelitis virus, or TMEV, causes a variety of neurological sequelae in rodents depending on the genetic background of the host. TMEV infection has long been used as a model for virally induced demyelinating disease or epilepsy, but recent studies in our lab have revealed outcomes to TMEV infection much more nuanced and complex than any previously seen in mice. These outcomes are more similar to the range of effects seen in humans with viral infections. To characterize the spectrum of responses to TMEV, we use the Collaborative Cross, a resource of diverse mouse strains derived from a crossbreeding scheme including five common (A/J, C57BL/6J, 129S1/SvlmJ, NOD/ShiLtJ, NZO/HlLtJ) and three wild-derived (CAST/EiJ, PWK/Ph, and WSB/EiJ) inbred mouse strains. This crossbreeding “funnel” renders each CC strain genetically and phenotypically distinct, with the genetic diversity of an outbred population but the reproducibility of an inbred population [1,2]. We have also evaluated CC-RIX strains (recombinant inbred intercrosses) as additional sources of diversity [3].

Previous studies have identified genetic factors linked to TMEV resistance or susceptibility, with these responses defined in relation to TMEV-induced demyelinating disease or viral persistence [4,5,6,7,8,9,10,11,12,13,14,15,16,17,18,19,20]. In fact, TMEV infection outcome has been studied in one of the eight CC founder strains: the TMEV-resistant strain C57BL/6J. However, the complex genetic diversity among the CC mouse strains has allowed us not only to identify novel TMEV-induced phenotypes, but to identify and explore additional genetic factors contributing to these responses. We hypothesized that genetic factors also underlie novel outcomes of TMEV infection, particularly resilience.

By evaluating long-term TMEV infection in 19 CC strains, we observed outcomes ranging from seizures to weakness and paralysis. Similar to humans infected by a virus, each individual CC strain responded uniquely to TMEV infection. We did not find TMEV persistence to be a driving factor for disease severity in any phenotype evaluated. We also observed TMEV outcomes unlike any previously described in conventional mouse strains. In addition to classical TMEV resistance (defined here as evidence of TMEV clearance with mild clinical phenotypes in the chronic phase of infection), and susceptibility (evidence of TMEV persistence with severe clinical phenotypes during chronic phase), we also identified several CC strains with persistent TMEV infection but mild clinical signs of disease during the late chronic phase. We define such mice as “resilient” to TMEV infection.

In the current study, we performed RNA sequencing during the late chronic phase of TMEV infection to identify key factors determining the severity of neurological symptoms. We first evaluated gene expression in all TMEV-infected mice versus sham-infected mice, pooling all 19 CC strains to understand the overall effect of TMEV infection on gene expression, host genetic backgrounds notwithstanding. Next, we categorized individual CC strains based on similar TMEV responses (resistant, resilient, or susceptible), and characterized the genetic underpinnings distinguishing each category.

By comparing differentially expressed genes (DEGs) across different TMEV response categories, we identified genes and sequence variants which correlate with TMEV resistance and susceptibility, and—most importantly—with resilience. We also identified novel biomarkers for TMEV disease outcomes. These findings provide additional context for understanding neurological dysfunction as a consequence of viral infection. Critically, these findings also elucidate *resilience* as an outcome to persistent viral infection, provide targets for mechanistic investigations, and expand our understanding of TMEV infection as a model for human neurological diseases.

## 2. Results

### 2.1. Expression of Gm41561, a Long Non-Coding RNA Gene, Was Significantly Affected by TMEV Infection Regardless of Mouse Strain

We compared differentially expressed genes between TMEV-infected and sham-infected mice from all CC strains in this study (Supplementary Table S1). Expression of only one gene, *Gm41561*, was significantly different between all infected and uninfected mice, with a log2 fold change value of −17.796. This predicted gene is on mouse chromosome 17 at 31,067,410–31,076,410 base pairs (based on Ensembl annotation of GRCm39), and encodes a long non-coding RNA (lncRNA). Although lncRNAs, including *Gm41561*, tend not to be conserved across species [21], this finding underscores the possibility of similar non-protein-coding loci being commonly involved in human viral infections.

*Gm41561* is located approximately 300 kb upstream of the H2 region. This proximity suggests potential linkage between *Gm41561* and H2, which has previously been associated with TMEV susceptibility [6,8,9]. *Gm41561* contained two exons and 815 variant alleles in mice. Any of these variant alleles could have downstream functional consequences; for example, lncRNA splice variants can produce myriad wide-ranging effects as they influence the expression and regulation of multiple genes and their downstream interactants [21]. We found no variants that correlated with the presence or absence of TMEV for any CC strain. However, 949 target genes have been identified as being differentially expressed in adipocytes following knockdown of *Gm41561* [22]. This finding reinforces the likelihood that *Gm41561* extensively affects gene expression differences in infected vs. sham-infected mice.

Comparing gene expression between pooled infected vs. pooled sham-infected mice was important for determining universal effects of TMEV infection on gene expression, but obscured the effects of the genetic diversity on viral response represented by the 19 CC strains. Therefore, we next compared infected and sham-infected mice from each strain separately, then grouped the strains based on similar phenotype profiles to identify molecular drivers of each profile.

### 2.2. CC Strains Demonstrated Novel Responses to TMEV Based on TMEV Persistence and Phenotypic Severity

**Figure 1:**
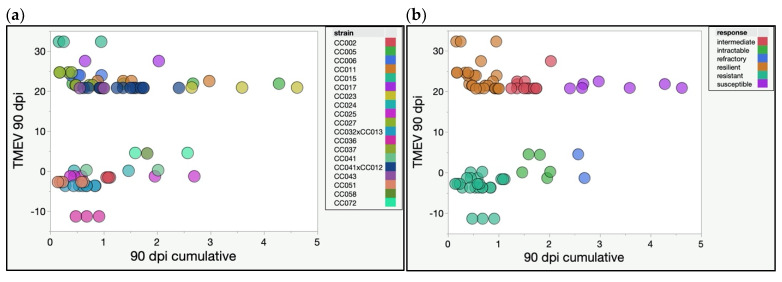
The 90 dpi cumulative scores (cumulative frequencies of hunching, delayed righting reflex, paresis, paralysis, clonus, ruffling, and encephalitis observed over 90 dpi, as described previously [23]) and TMEV RNA levels (shown as log2FoldChange values) at 90 dpi were plotted with different colors for each CC strain (**a**) or response category (**b**).

We identified distinct TMEV response profiles by overlaying 90 dpi cumulative phenotype scores, and levels of TMEV RNA measured at 90 dpi (Figure 1). The cumulative phenotype scores are the sum of the scores for multiple phenotypes and therefore represent the totality of TMEV phenotypes over time. These scores were used to compare levels of phenotypic severity experienced by different CC strains.

**Figure 2:**
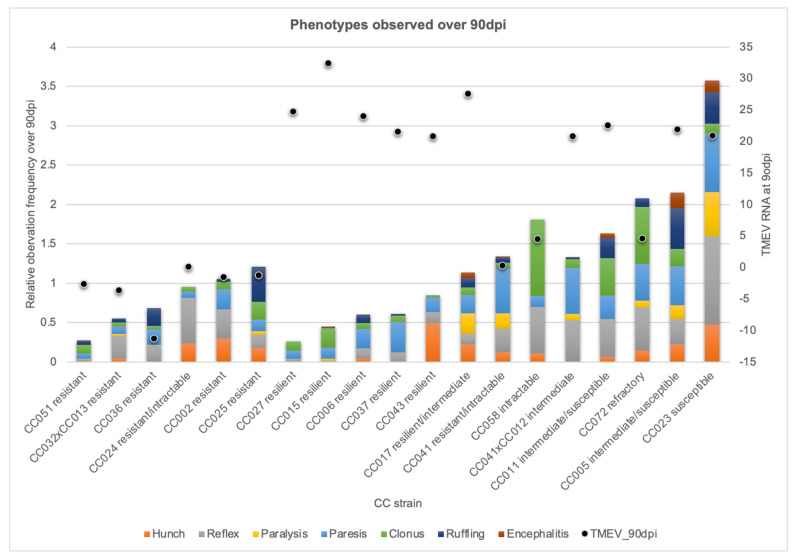
Full 90 dpi cumulative phenotype profiles for each strain are shown with each of the 7 phenotype categories distinguished by color. Strains are ordered from left to right by response category. “Relative observation frequency over 90 dpi” shows the cumulative frequencies of hunching, delayed righting reflex, paresis, paralysis, clonus, ruffling, and encephalitis observed over 90 dpi, as described previously [23]. “TMEV RNA at 90 dpi” shows levels of TMEV RNA measured at 90 dpi (log2FoldChange values) as dots.

Infected mice of the same strain had consistent phenotype profiles, demonstrating phenotypic reproducibility. We also found that certain strains consistently clustered together. These clusters represented different TMEV response profiles. Importantly, TMEV persistence, as measured by TMEV RNA levels, did not correlate with phenotype severity, even when considering each of the seven phenotypic classes separately (Figure 2).

Strains with little to no detectable TMEV RNA at 90 dpi had correspondingly low phenotype scores. We termed such strains “resistant” based on the TMEV response described previously for TMEV-“resistant” C57BL/6J mice [20,24]. Resistant strains included CC002, CC032×CC013, CC036, and CC051.

We identified other mouse strains with low cumulative 90 dpi scores, similar to resistant strains, but with high levels of TMEV RNA measured at 90 dpi. The high viral presence and low phenotype scores indicated these strains tolerated the ongoing infection. We called these strains “resilient” because not only did these mice tolerate the virus without succumbing, but they also showed minimal signs of suffering from disease. Resilient strains included CC006, CC015, CC027, CC037, and CC043.

On the other hand, some strains exhibited moderately high cumulative 90 dpi scores, but virtually no TMEV RNA was detected at 90 dpi. We considered these mice to have “intractable” disease, as the cause of the disease symptoms (TMEV) had been effectively eliminated but the symptoms continued to persist. We considered the most severe intractable cases to be “refractory” because cumulative scores in these strains were among the highest measured, despite low levels of TMEV RNA. Mice of the strain CC072 fell into this category.

Strains with “intermediate” TMEV susceptibility have been described previously [6,25]. Such mice had persistent TMEV infection with moderate cumulative phenotype scores. All but one mouse from strain CC041×CC012 demonstrated an intermediate TMEV response.

Finally, strains with high levels of TMEV RNA and high 90 dpi scores were classified as “susceptible.” Susceptible mice in this study included strain CC023. These mice experienced the most severe and debilitating of the TMEV-induced neurological deficits while continuing to show signs of persistent infection.

Mice from other strains included in this study did not fall consistently into a single response category. We could not conclude whether sex played a role in response differences in these strains, due to small numbers of mice per sex/strain combinations. However, individual mice within each of these strains exhibited similar TMEV responses. We therefore classified these strains as follows: CC005 and CC011—intermediate/susceptible; CC017—resilient/intermediate; CC024 females—intractable, CC024 males—resistant; CC025—all but one mouse scored as resistant; CC041 females—resistant, CC041 males—intractable; CC058—intractable. 

### 2.3. Genetic Diversity Contributed to Protection, Compensation, or Capitulation in the Face of TMEV Infection

We used Ingenuity Pathways Analysis to better understand the overall influence of TMEV infection (Table 1). This analysis included all statistically significant DEGs for all strains. Two top Canonical Pathways were identified: Neuroinflammation Signaling Pathway and GABA Receptor Signaling. Each pathway has been implicated in neurodegenerative diseases (e.g., [26]) and viral infections (e.g., [27]).

We next tested the hypothesis that different TMEV response profiles were associated with distinct gene expression profiles. For this, we developed gene expression profiles for each strain using the Analyses feature of IPA. We then used the Comparison Analysis feature of IPA to compare CC strains within each TMEV response category and identify similarly affected networks, biological functions, and canonical pathways.

**Figure 3:**
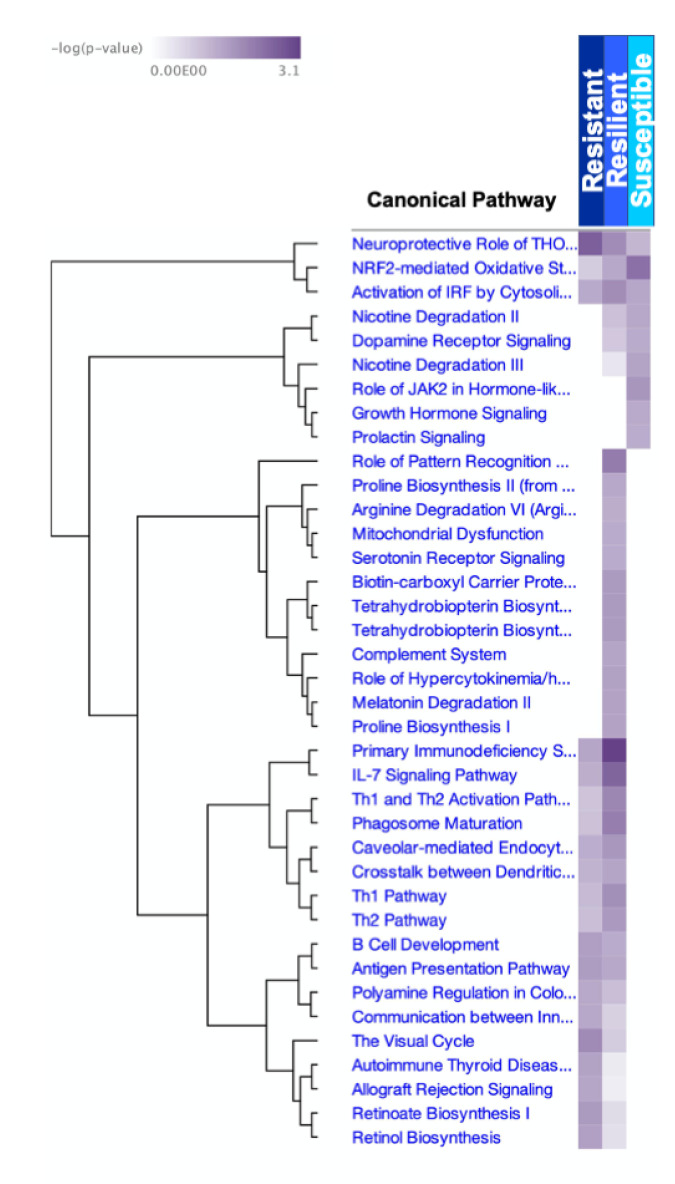
Significant Canonical Pathways are listed for each response group. The significance of each pathway to each response group is shown as –log (*p*-value), with darker shading indicating greater significance.

Canonical Pathways analyses included statistically significant DEGs for strains of the resistant, resilient, and susceptible TMEV response categories. Top Canonical Pathways varied by response group, with some pathways shared between groups as well (Figure 3). The pathway “Activation of IRF by cytosolic pattern recognition receptors” was significantly affected across all groups, suggesting that all mice recognized the presence of the virus, irrespective of their different responses. For resistant strains the most significant Canonical Pathway was “Neuroprotective Role of THOP1 [Thimet oligopeptidase] in Alzheimer’s Disease” (−log *p* = 2.59). This same pathway was also significant, though to a lesser degree, for resilient (−log *p* = 1.87) and susceptible (−log *p* = 1.19) strains. In fact, no significant canonical pathways were unique to the resistant response profile. This indicates “resistance” was based on the relative degree to which certain pathways (or the genes involved) were affected.

The top Canonical Pathway for resilient strains was “Primary Immunodeficiency Signaling” (−log *p* = 3.08), which was also significant in resistant strains (−log *p* = 1.46) but not susceptible strains. Additionally, 12 Canonical Pathways were significant only for resilient strains. The most significant of these was “Role of Pattern Recognition Receptors in Recognition of Bacteria and Viruses” (−log *p* = 2.12).

“NRF2-mediated Oxidative Stress Response” was the top Canonical Pathway for the susceptible category (−log *p* = 2.30). This pathway was also significant in resilient (−log *p* = 1.42) strains, though to a lesser extent. Three significant pathways were unique to the susceptible response category: “Role of JAK2 in Hormone-like Cytokine Signaling” (−log *p* = 1.72), “Growth Hormone Signaling” (−log *p* = 1.40), and “Prolactin Signaling” (−log *p* = 1.34). The same molecule, prolactin (PRL), was involved in all three of these pathways. PRL was not involved in pathways related to resistant or resilient responses.

We next performed gene network analyses to identify networks connecting gene expression differences with biological functions and diseases. These analyses demonstrated how the roles of specific molecules were influenced by overall host genetic background. We identified top networks for all strains together (“overall”), and for resistant, resilient, and susceptible strains separately (Supplementary Table S2). We then investigated the diseases and biological functions that could be expected to be significantly affected by TMEV infection, based on genes in top networks (Supplementary Table S3), using the IPA “Downstream Effects Analysis.” 

Networks for the overall group all had scores of three, indicating these networks had low chances of potential causal relevance (for more details about IPA scoring, refer to [29]). Nevertheless, the molecules in those networks had functions known to be perturbed in other viral infections of the CNS. For example, TBX19 is involved in the accumulation of progenitor cells; reduced proliferation of neural stem/progenitor cells and impaired adult neurogenesis have also been observed in herpes simplex 1 infection [30]. Another function of TBX19 potentially affected by TMEV infection was “Development of pituitary gland;” pituitary dysfunction following acute viral meningoencephalitis (e.g., [31,32], reviewed in [33]) and viral meningitis (e.g., [34]) have been reported. Despite the low network scores, evidence suggested that TMEV-induced perturbations in gene expression could affect developmental and endocrinological biological functions, along with immune and neurological functions.

Next, we identified the networks and diseases/biological functions affected by TMEV for each response group. The top network for resistant strains (score of 27) is related to biological functions generally involving repair and regulating cytotoxic immune responses. Many top networks were listed for resilient strains, the highest with a score of 41; many functions associated with these networks pertain to inflammation and innate immune response as well as development and cell cycle regulation. For the susceptible category, functions related to the single network (score of 46) involve hormone-sensitive responses and regulation which collectively affect cell signaling and cell cycle. Among biological functions affected by these networks, “Small Molecule Biochemistry” was the only one shared by all categories. However, this function is listed in different contexts for different categories: for resistant strains, the same network that affects “Small Molecule Biochemistry” also affects “Energy Production” and “Lipid Metabolism.” In resilient strains, the same network affecting “Small Molecule Biochemistry” also affects “Cell-To-Cell Signaling and Interaction” and “Humoral Immune Response;” for susceptible strains, “Cell Signaling” and “Cell Cycle” are affected by the same network as “Small Molecule Biochemistry.” Only one gene, peptidylprolyl isomerase B (*Ppib*), was listed for resistant, resilient, and susceptible TMEV response groups under the category “Small Molecule Biochemistry” (Supplementary Table S3); in each case, the role of *Ppib* was related to cytotoxicity.

To identify common effects of TMEV infection that manifested differently depending on context, we characterized the molecules in each network (including genes and complexes) which effected biological functions across multiple response groups. We noted 37 molecules found in >1 networks. Of these molecules, 15 were found in networks for both resistant and resilient strains, 13 for resilient and susceptible, 2 for resistant and susceptible, and 5 were included in networks for all three response groups. Additionally, one gene (*Igkv4-61*) was found in networks for susceptible and overall, and one complex (MHC class II) in networks for resilient, susceptible, and overall. In resilient strains, three networks included the Il-12 complex. These findings reflect the multiple roles played by each gene/complex, roles which vary based on the broader “expression context” of a given TMEV response category.

### 2.4. Upstream Regulators of Biological Functions and Their Molecular Targets, Varied by TMEV Response Group

For each of the different TMEV response groups, we identified the top five Upstream Regulators (URs), specific genes with expression connected to the biological functional categories influenced by the networks/molecules in Supplementary Tables S2 and S3 (Table 2). Most (four out of five) of the URs associated with the “Overall” group regulate transcription; the UR miR-122-5p is a microRNA known to regulate antiviral responses in humans, particularly in hepatitis C infection (e.g., [35,36]). The target of regulator NFIA (nuclear factor I A), GABRA6 (gamma-aminobutyric acid receptor subunit alpha-6), interacts with the inhibitory neurotransmitter GABA.

The Resistant and Resilient groups shared only one UR in common (MSH2, mutS homolog 2), but the molecules targeted by the URs of these two groups overlapped somewhat. Only one target molecule differed in the Resistant group compared to Resilient: *Ccl6* (chemokine [C-C motif] ligand 6), a type of small cytokine only found in rodents. Other URs for the Resistant group had well-known, multifaceted roles in controlling the immune response. For example, C-X-C motif chemokine ligand 10 (CXCL10) also stimulates multiple types of immune cells and has known roles in neuronal injury related to viral infection and in relevant human disorders such as multiple sclerosis [38]. Retinoic acid early transcripts 1D/1E (*Raet1d*/*Raet1e*), related to major histocompatibility complex class I genes, are part of a family of glycoproteins involved in immune responses and expressed in pathological conditions, notably experimental autoimmune encephalomyelitis in mice [39] and mouse cytomegalovirus [40]. The “outlier” of the URs for the Resistant group was heat shock protein 990 (HSP-990), a synthetic HSP90 inhibitor with potential therapeutic use in cancer treatment [41]. Hsp90 has been identified as an important host factor in the life cycle of TMEV [42]: Hsp90 colocalizes with the VP1 subunit of TMEV during infection [43].

URs for the Resilient group, aside from MSH2, included polynucleotide phosphorylase (PNPT1), an enzyme which has been associated with a spectrum of neurodegenerative phenotypes (e.g., [44,45]). Another UR encodes immunity-related GTPase family M protein 1 (*Irgm1*), which modulates resistance to pathogens [46,47] and can contribute to autoimmunity [48,49]; similarly, the UR interferon beta 1 (IFNB1) is crucial for the antiviral immune response but can also contribute to autoimmunity (reviewed in [50,51]). Finally, the UR ELAV-like RNA binding protein 1 (ELAVL1) functions in regulating the innate immune response via its RNA binding capabilities [52,53]. Together, these URs targeted many more molecules in addition to those listed for the Resistant group. The functions of these UR target molecules gave some insight into the molecular differences distinguishing the responses of the resilient strains. Other targets contributing to the antiviral response included interferon gamma inducible protein 16 (IFI16), which mediates interferon beta production in response to viral infection [54], and interferon induced with helicase C domain 1 (IFIH1), which senses viral RNA to provoke an antiviral immune response and occasionally contributes to autoimmune diseases (for example, [55]). The protein encoded by the target gene 2′–5′ oligoadenylate synthetase 1B (*Oas1b*) was found to affect susceptibility to West Nile Virus in CC mice [56]. The target molecules *Apol9a/Apol9b* function to inhibit TMEV replication [57]. Variants of target molecules GBP3 and GBP6 (guanylate binding proteins 3 and 6) have antiviral activity (e.g., [58]).

Susceptible group URs included guanine nucleotide-binding protein, alpha subunit (GNAS), an imprinted (i.e., methylation-regulated) locus with a complex expression pattern. GNAS is implicated in the production and function of hormones that regulate endocrine glands, such as the pituitary gland and thyroid, along with ovaries and testis. Another UR identified was the compound BIM-23A760, a chimeric somatostatin/dopamine agent used for controlling proliferation of non-functioning pituitary adenomas [59,60,61]. The IQ motif and ubiquitin domain-containing protein may contribute to cell proliferation and migration by activating the Akt/GSK3β/β-catenin signaling pathway [62]. The UR Ras homolog family member Q (RHOQ) has an important function in nerve regeneration/elongation [63,64] and a role in physiological B cell responses [65]. Finally, the UR ubiquitin conjugating enzyme E2 Q1 (UBE2Q1) is regulated via methylation and functions to flag proteins for degradation by modifying them with ubiquitin [66]. UBE2Q1 is a potential biomarker for hepatocellular carcinoma [67,68,69] and ovarian cancer [70], and may also function in female hormone homeostasis (for example, [71]). All five top URs for the susceptible group target prolactin (PRL); GNAS also targets growth differentiation factor 9 (GDF9). GDF9 regulates ovarian function [72,73]. PRL, meanwhile, is also a growth regulator, including for the immune system (for example, [74,75]).

### 2.5. Unique Biomarkers Distinguished TMEV Response Categories

We identified known and novel molecular biomarker candidates for each CC strain using the Biomarker Filter Results feature of IPA, filtering by species (mouse), tissues (nervous system), and *p*-adj value equal or less than 0.05 (except for the overall group, where the average *p*-adj value was 0.999). We then used the Biomarker Comparison Analyses feature to compare biomarker differences and similarities across different TMEV response profiles, and to identify unique biomarkers for each (Table 3).

For the overall group, we performed a Biomarker Filter Analysis to identify potential biomarkers which could reveal common molecular contributors to TMEV’s overall effects across multiple genetically diverse strains. One biomarker resulted from this analysis: staufen double-stranded RNA binding protein 1 (STAU1). STAU1 has been found to promote viral replication in several types of viral infections (for example: influenza [76], human immunodeficiency virus type 1 [HIV-1, [77]], human endogenous retrovirus [HERV-K, [78]], and Ebola [79]). Expression levels for *Stau1* were all low in infected vs. sham-infected mice, and varied little across all strains in this study (Supplementary Table S1).

The sole biomarker for the resistant group, HLA-A, appeared in the top-scoring networks of both resistant and resilient groups (Supplementary Table S2). However, it was listed as a molecule relevant to diseases and biological functions far more often for the Resistant group (32 vs. 8; Supplementary Table S3). HLA-A is not a mouse gene; rather, this class I gene in the major histocompatibility complex of humans is homologous to several class I genes in mice (*H2-D1*, -*K1*, -*Bl*, -*Q1*, -*Q2*, -*Q4*, -*Q6*, and -*Q10*).

The two biomarkers for the resilient group, *Cdpf1* and *Fgf4*, offered insight into what distinguished the resilient strains from other TMEV responses. The biological functions with which these biomarkers were associated included cancers (i.e., cell growth, transformation, and survival), cell signaling (including binding and adhesion of blood cells and myelosuppression, all likely related to inflammation), and infectious diseases (binding of viruses). Other related functions included tissue morphology, molecular transport, and protein phosphorylation.

Gene expression levels (Table 3, Expr Log Ratio column) for biomarkers of the susceptible response were markedly lower than for any other strain. Biomarker EIF3J (*Eif3j2* in mice) had roles in protein synthesis, metabolism, and translation, sharing these networks with another biomarker, *Mid1*. *Mid1* was associated with nervous system development in the susceptible category, but for resilient strains *Mid1* was more often found in networks related to innate immune response and inflammation. *Gm5148* was represented in only one network, with the biofunction “phosphorylation of protein,” listed with the resilient category despite *Gm5148* being a biomarker for susceptible response. *Gm5148* was listed with *Rps23rg1*, a mouse gene on a different chromosome and having different functions altogether; however, *Rps23rg1* was not included in any networks for any groups.

The two remaining biomarkers, *Gdf9* and *Prl*, were found only in networks listed for the susceptible response category. Their biological functions in this context were primarily endocrine-related.

### 2.6. Haplotypes Provided Context for Pleiotropy and Predictive Alleles

Founder haplotypes (alleles) were identified for 89 genes that effected biological functions relevant to TMEV responses, including genes present in multiple networks, genes present in canonical pathways, URs, UR target molecules, and biomarkers (Supplementary Table S4). Increased heterozygosity of these genes was often associated with resistant or resilient TMEV responses: 13 out of 89 genes were heterozygous in two (out of four) resistant strains, and one gene (*Sfi1*) was heterozygous in three resistant strains. Five genes were also heterozygous in two (out of five) resilient strains. In the susceptible category, only two genes were heterozygous. Of particular interest was the HLA-A region, due to its historical context with TMEV infection: resistant strains CC032×CC013 and CC015, and resilient strains CC006 and CC027, were heterozygous for the HLA-A region.

Strains from resistant, resilient, and susceptible groups shared the same homozygous founder haplotype for nine genes (Supplementary Table S4). However, it was more common for strains of the same response group to share haplotypes with one another than with strains of other response groups. For example, in the four strains of the resistant group, seven out of the eight possible alleles for *Tmem203* were inherited from the founder strain 129S1/SvImJ; the 129S1/SvImJ haplotype was not found in resilient or susceptible strains. For the gene *Nnmt*, eight of ten founder alleles for the resilient group were inherited from the founder strain WSB/EiJ; the WSB/EiJ haplotype was not present in other strains. For resistant mice, the majority of alleles for six genes were inherited from a founder strain not represented in the alleles for resilient and susceptible mice. Similarly, the predominant alleles of eight genes were found only in resilient strains. The susceptible strain CC023 shared the fewest haplotypes with other strains and groups: there were 21 genes with CC023-specific founder alleles. One surprising exception was the HLA-A region, for which resistant strain CC002 and susceptible strain CC023 shared the same haplotype, inherited from 129S1/SvImJ.

To determine the possible functional relevance of response group-specific haplotypes, we identified sequence variants inherited from each founder strain for those genes with the highest response-specific allele frequencies: *Tmem203* for resistant strains, and *Nnmt* for resilient strains. We found no SNPs or sequence variants unique to the 129S1/SvImJ founder strain from which most resistant mice inherited *Tmem203*. However, we identified SNPs and sequence variants for *Nnmt* that were unique to WSB/EiJ, the most common founder allele for this gene in resilient strains. Two of these variants were classified as transcription factor-binding site variants (SNPs rs263473586 and rs1132394264), located 8bp from each other upstream of *Nnmt*. Both of these variants were located within a CTCF binding site (ENSMUSR00000747534) associated with regulatory action in the developing mouse brain. In our search for *Nnmt* variants we also uncovered 19 additional SNPs and two indels, which were identified as upstream gene variants associated with miRNA ENSMUSG00002076361. We next identified a *Nnmt* sequence variation specific to the A/J founder strain, relevant to susceptible strain CC023 (and intermediate/susceptible strain CC011). The only unique and potentially functionally relevant sequence variation identified for the A/J strain was SNP rs1134607613, an upstream gene variant associated with miRNA ENSMUSG00002076361, and located farther upstream than the WSB/EiJ variants. We did not measure miRNA expression in this study and therefore could not evaluate how these variants influenced expression of ENSMUSG00002076361. However, because all these variants were upstream of the pairing region of the miRNA, it is reasonable to expect these variants could affect its production [80,81]. ENSMUSG00002076361 was not listed in miRBase [82], but a sequence comparison (blastn) of its sequence revealed similar sequences present on at least 11 other chromosomes. MiRNAs can have pleiotropic effects in that they can regulate multiple genes [80]. Therefore, these similar sequences could reflect targets of this miRNA.

## 3. Discussion

In this study using genetically diverse mouse strains, we evaluated interactions between DEGs and how these interactions contributed to different long-term outcomes to TMEV infection. By comparing gene expression profiles in TMEV-infected and control mice of the same strain, we reduced the background “noise” and focused only on the effects of TMEV infection in each strain. The TMEV response profiles produced with this approach allowed us to associate significant DEGs with TMEV response (phenotype severity). In doing so, we identified a novel response, “resilience,” characterized by relatively mild symptom profiles with high levels of TMEV RNA. This contrasts with the current paradigm of TMEV infection, wherein strains considered “susceptible” to persistent TMEV infection develop demyelinating disease and “resistant” strains clear the infection and experience seizures. While such clear-cut distinctions are helpful for, e.g., mechanistic studies of demyelination, human outcomes to viral infection tend to be far more nuanced.

Comparisons of DEGs among individual strains, even between TMEV response groups, revealed few strong correlations between gene expression and TMEV outcome. For most of the 89 genes that were the focus of this study, expression levels differed little among strains (Supplementary Table S1). We found it more appropriate to generate response-specific expression profiles, placing individual genes in context of pathways and networks.

As expected, we found resistant mouse strains showed evidence of an appropriate and effective immune response mediated by the major histocompatibility complex class I region. The top Canonical Pathway for resistant strains, “Neuroprotective role of THOP1 in Alzheimer’s Disease,” is associated with enhanced protection against neurodegeneration [83,84] and enrichment of this pathway in the resistant strains may explain the mild neurological symptoms observed in these mice. The sole biomarker for the resistant group, HLA-A, has a critical role in the immune system and, by extension, responses to infectious agents such as viruses. Expression levels of the mouse homologs of HLA-A did not correlate directly with TMEV response; however, the mouse HLA-A homologs have thousands of polymorphic alleles—sequence variants with cumulative effects on immune response. The role of H2 class I alleles in TMEV infection has been described for inbred mouse strains [6,8,9] and for the CC strains included in this study [23,85]. Though the H2 class I region was inherited from the same founder strains for some CC strains of different response categories, interactions between the HLA-A homologs and other genes within the same networks influenced the TMEV-resistant outcome.

Resilient strains failed to eliminate the viral infection but moderated its effects, possibly by disabling the virus or reducing its virulence, for example by inhibiting TMEV replication or enhancing RNA degradation. Members of the top Canonical Pathway for resilient strains, “Primary Immunodeficiency Signaling,” can provide protection against immune depletion while inhibiting viral spreading. Differential expression of pathway molecules may therefore serve in a compensatory fashion for the resilient strains as these mice maintain a relatively heavy viral load while experiencing minimal symptoms. However, primary immunodeficiency often coincides with/causes autoimmunity [86,87,88,89,90]. This seemingly paradoxical co-occurrence—a deficient immune response coupled with a powerful immune response—results from complex interactions between different signaling pathways, and is the product of hereditary factors [87]. While a deficient immune response could help explain the relatively high levels of TMEV RNA measured in resilient strains at 90 dpi, other pathways collaborated to ensure these mice continued to live relatively symptom-free lives. Among the pathways significant only for resilient strains, the most significant was “Role of Pattern Recognition Receptors in Recognition of Bacteria and Viruses.” Different classes of germline-encoded pattern recognition receptors can recognize pathogens and trigger innate and adaptive immune responses (reviewed in [91,92]). Genetic differences contribute to the relative effectiveness of innate immune responses to TMEV infection, as described for resistant and susceptible inbred strains (e.g., [93,94]). Different substrains of BALB/c mice exhibit varying levels of susceptibility to TMEV-induced demyelination, including an “intermediate” response [95], so there is precedent for a response to TMEV that is neither resistant nor susceptible; however, to our knowledge the current study is the first to characterize a resilient response to TMEV infection. The resilient strains may be able to control the virus to a level whereby it could no longer cause damage, but may still persist. Though resilient strains retained TMEV RNA into the late chronic phase of infection, these mice survived and maintained biological functions, implicating crucial roles for non-immune networks and molecules. Resilient strain networks included categories of molecules containing both biomarkers *Cdpf1* and *Fgf4* with HLA-A. These categories/molecules were associated with cancers and with organismal injury and abnormalities (Supplementary Table S2). Further, the resilient group biomarker FGF4 promotes stemness and proliferation of human stem cells [96,97]. Taken together, our findings suggest a balanced relationship between cell growth and survival, and immune response/recognition, in the resilient strains.

The most susceptible group was characterized by a relative paucity of immune-related pathways and regulators. Instead, the response of the susceptible mice appears to have been hampered by endocrine-related factors. The susceptible strain biomarkers indicated the environment in which these mice failed to thrive in the face of TMEV infection. *Rps23rg1* has been associated with reduced beta-amyloid levels in Alzheimer’s disease [98,99]; deletion of this gene decreases synaptic integrity and function [100]. Next, *Mid1* is normally strongly upregulated in murine cytotoxic lymphocytes and plays a role in granule exocytosis [101]. In humans infected with rhinovirus, Mid1 is normally upregulated in bronchial epithelial cells, suggesting a link to innate immune pathway activation and inflammation [102]. Finally, the *Prl* gene has multiple roles relevant to TMEV outcomes: *Prl* can stimulate cells of adaptive and innate immune responses [103], is neuroprotective, and has promyelinating properties [104,105,106]. Therefore, prolactin could have a beneficial effect on the neurological and immunological outcomes of TMEV infection. However, *Prl* expression levels were very significantly decreased for infected susceptible mice compared to uninfected mice (fold change −17.432). The top URs for the susceptible group all decreased prolactin gene expression. This may indicate a response meant to counter another, harmful effect of prolactin: inflammation that leads to autoimmunity [107,108,109,110,111,112]. Further mechanistic studies are needed to better understand the roles of *Prl* in relation to TMEV infection and neuropathology.

Haplotype and allelic variation demonstrated how the genetic diversity of the CC strains contributed to the phenotypic diversity underlying the different TMEV response groups. We identified sequence variants with potential functional relevancy by identifying genes (from the list of 89 genes of interest) for which a single founder strain was the most common source of alleles for strains of a specific TMEV response group. We have in this way identified potential targets for future mechanistic studies. In particular, the strong association of *Nnmt* haplotypes with the resilient TMEV response group suggested a potential role for this gene in resilience.

In the present study, we compared sequence variation across the CC founder strains with a focus on those variants specific to the WSB/EiJ founder (associated with the resilient response group for *Nnmt*) and A/J founder (associated with the susceptible group for *Nnmt*). *Nnmt* has been associated with neurodegeneration and Parkinsonian behavior in humans [113,114] and the model organism *C. elegans* [115], and more recently with Alzheimer’s disease [116]. Dysregulation of *Nnmt* is recognized as a contributor to neurological diseases, cancers, and obesity (e.g., [116,117,118,119,120]). In fact, at least one *Nnmt* sequence variant has been connected with neurological disease in humans [119]. Interestingly, *Nnmt* has also been shown to have neuroprotective effects [121]. Most of the resilience-associated SNPs and indels we identified within the *Nnmt* gene were synonymous SNPs unlikely to contribute to functional differences. Two variants, however, were associated with a regulatory element: a CTCF binding site. The CTCF zinc finger protein has myriad genetic and epigenetic regulatory capabilities, and plays numerous functional roles via its capacity for context-dependent (“custom”) gene regulation [122,123,124,125]. We did not find any *Nnmt* sequence variants with functional relevance for the susceptible response.

Our search for sequence variation relevant to *Nnmt* also uncovered variants associated with a miRNA gene located in close proximity to *Nnmt*. Resilient strains contained 19 such variants; susceptible mice had one variant. These miRNA-associated variants could influence the production of the miRNA and, by extension, its regulatory capacity [126]. The regulatory function of miRNAs results in pleiotropy: basically, a single gene influencing the expression of many other genes [80]. SNPs affecting miRNAs are implicated in neurological conditions (reviewed in [127,128,129]), and many other complex diseases [130]. Furthermore, recent studies describe miRNA links between Epstein–Barr virus and multiple sclerosis [131,132,133], adding plausibility to the idea of miRNA involvement in TMEV response. In fact, miR-219 has been associated with reduced TMEV replication and TMEV-induced demyelination [134], though TMEV itself does not appear to be a target of miRNAs [135].

Taken together, our findings suggest that variations specific to the genetic background of the host interact with the rest of the genome in a “domino effect” resulting in different categories of TMEV response. While one path of this “domino effect” leads to TMEV clearance or persistence, the next path can lead to symptoms that persist or worsen (susceptibility) or improve or even appear to resolve entirely (resilience). Smaller “branches” off these different paths lead to minor nuances in TMEV outcome, such as TMEV-induced symptoms that remain intractable even once the virus is cleared. The larger pathways and networks involved in the broader TMEV outcomes (resistant, resilient, and susceptible) provide targets for future studies to reveal the mechanisms underlying different responses to TMEV.

## 4. Materials and Methods

### 4.1. Mice and Phenotyping

Ethics statement: All procedures were approved by the Institutional Animal Care and Use Committee at Texas A&M University and performed under animal use protocol numbers 2017-0082 (approved 20 July, 2017) and 2020-0065 (approved 21 May, 2020). All experiments were performed in accordance with relevant guidelines and regulations. Mice were group-housed and all testing performed during the light phase.

As described in [23], at 4 weeks of age, female and male mice were anesthetized by isoflurane inhalation (MWI, Meridian, ID, USA) and injected intracerebrally with 5.0 × 10^4^ plaque-forming units (PFU) of the BeAn strain of TMEV (American Type Culture Collection [ATCC] VR 995, Manassas, VA, USA) in 20 μL of PBS placed into the fenestra at a depth of approximately 1.5 mm [136,137]. Sham-infected mice (*n* = 25 females and 27 males) were anesthetized and injected with PBS only. We used the “cumulative phenotype score (90 dpi score)” as defined in [23] to quantitatively compare TMEV outcomes across strains. Briefly, multiple phenotype classes were scored daily during the acute phase of infection (0–14dpi) and weekly thereafter (15–90 dpi). These classes included hunching, righting reflex, paralysis, paresis (weakness), clonus, ruffling (piloerection), and encephalitis, detailed in [23]. The sum of the scores for these phenotypes was the “cumulative phenotype score,” called “90 dpi score,” as the value reflects the frequency of observation for multiple neurological phenotypes over 90 dpi. 

Numbers of mice of each sex and infection status for each strain, along with the average TMEV RNA levels measured at 90 dpi, and average cumulative scores at 90 dpi, are shown in Table 4. Whenever possible, littermates were used to avoid batch effects within a strain.

### 4.2. RNA Isolation and Sequencing

RNA was isolated from hippocampi and thoracic spinal cords of 145 mice of 19 CC mouse strains (see Table 1 for details) and quantified with the Qubit Fluorometer (Life Technologies, Carlsbad, CA, USA) with a broad range RNA assay. Concentrations were normalized for library preparation, and RNA quality was verified on the Agilent TapeStation with RNA ScreenTape. RNA of sufficient quantity and quality was not uniformly available for infected and uninfected mice of both sexes for all strains; therefore, RNA sequencing data reflect a mixture of the two tissues. While not ideal for understanding tissue-specific gene expression, this procedure nonetheless allowed an overview of gene expression changes related to TMEV infection. Details regarding the generation of RNA sequencing libraries and sequencing procedures, including downstream processing and quality control, have been reported previously [23]. To evaluate the relative persistence of TMEV at 90 dpi, we measured expression (Fold Change) of the polyprotein AAA47930.1 of the TMEV virus after DEG (Differentially Expressed Genes) test was calculated using DESeq2 based on infection state (i.e., infection is present) for each strain.

### 4.3. Identification of Key Pathways, Networks, and Regulatory Molecules

Ingenuity Pathways Analysis (IPA) software was used to evaluate gene expression data, identifying key networks and pathways for each individual strain, for all strains combined, and for groups of strains (specifically resistant, resilient, and susceptible response groups). The “overall” group included all mice in the study. For analyses specific to TMEV response groups, resistant strains included all mice from CC002, CC032×CC013, CC036, and CC051; resilient strains included all mice from CC006, CC015, CC027, CC037, and CC043; susceptible mice included all mice from strain CC023. Strains for which categorization varied by sex (e.g., CC024 and CC041), or TMEV response groups represented by all members of only one strain (e.g., intermediate [CC041×CC012], intractable [CC058], and refractory [CC072]), or strains which represented more than one response group (e.g., CC005, CC011, and CC017) were not included in response group-specific evaluations.

Target molecules regulated by the top genes and proteins governing each network/pathway were also identified. Biomarkers were identified for each response group using IPA’s Biomarker Filter function. 

IPA calculates *p*-values differently depending on the analysis, as described [28]. In general, significance was determined using Fisher’s Exact Test. We applied the Benjamini–Hochberg method for multiple testing correction when identifying significant Canonical Pathways, Upstream Regulators, Networks, and Diseases/Functions.

### 4.4. Haplotypes and Sequence Variation

Haplotypes for loci of interest were identified using the Collaborative Cross Viewer [138,139]. SNPs within these loci were identified by querying two separate datasets: Sanger4 (for CC founder strains) and UNC-GMUGA1 (for CC strains and founder strains) [140,141] via the Mouse Phenome Database (MPD) (RRID:SCR_003212) [142]. Additionally, the Mouse Genomes Project was queried for SNPs, insertion/deletion variants (indels), and structural variants within and near loci of interest for CC founder strain genomes [143,144].

## 5. Conclusions

This study revealed a novel outcome for TMEV infection: resilience, which has features of both resistance and susceptibility to infection. Gene expression analysis allowed the comparison of pathways and networks involved in different TMEV outcome categories, which were distinguished from each other by collecting phenotype data from 19 genetically diverse mouse strains over 90 days post-infection. Expression profiling of resistant, resilient, and susceptible mouse strains revealed functionally relevant genetic variation, such as sequence-level differences in non-coding RNAs and miRNAs, which modulate gene expression and interactivity.

## Figures and Tables

**Table 1:** Top 5 canonical pathways for categories described in this study, along with their respective *p*-values [28], and the key molecules (genes or complexes) involved in these pathways. Arrows indicate the direction of gene expression (increased or decreased) in infected versus uninfected mice, based on the averaged expression values for strains included in each response. Additional information for these molecules, including descriptions and strain-specific expression, is available in Supplementary Table S1.

Top 5 Canonical Pathways	*p*-Value	Molecules
**Overall**		
Neuroinflammation Signaling Pathway	1.32 × 10^−2^	GABRA6 ↓
GABA Receptor Signaling	2.94 × 10^−2^	GABRA6 ↓
**Resistant**		
Neuroprotective Role of THOP1 in Alzheimer’s Disease	2.59 × 10^−3^	HLA-A ↑, PRSS41 ↑
The Visual Cycle	1.29 × 10^−2^	RDH13 ↑
Retinoate Biosynthesis I	2.31 × 10^−2^	RDH13 ↑
Antigen Presentation Pathway	2.50 × 10^−2^	HLA-A ↑
B Cell Development	2.75 × 10^−2^	HLA-A ↑
**Resilient**		
Primary Immunodeficiency Signaling	8.23 × 10^−4^	CD4 ↑, Igha ↑, IGHG1 ↑, Ighg2b ↑
IL-7 Signaling Pathway	3.21 × 10^−3^	Igha ↑, IGHG1 ↑, Ighg2b ↑, Ighg2c ↑
Role of Pattern Recognition Receptors in Recognition of Bacteria and Viruses	7.53 × 10^−3^	IFIH1 ↑, IL25 ↑, LTA ↑, Oas1b ↑, Oas1d ↓ (includes others)
Phagosome Maturation	8.14 × 10^−3^	DYNLT1 ↑, HLA-A ↑, HLA-E ↑, PRDX1 ↑, TUBD1 ↑
Th1 and Th2 Activation Pathway	1.12 × 10^−2^	Aph1c ↓, CD4 ↑, HLA-A ↑, IL25 ↑, LTA ↑
**Susceptible**		
NRF2-mediated Oxidative Stress Response	5.01 × 10^−3^	AOX1 ↑, PPIB ↑
Role of JAK2 in Hormone-like Cytokine Signaling	1.92 × 10^−2^	PRL ↓
Nicotine Degradation III	3.20 × 10^−2^	AOX1 ↑
Activation of IRF by Cytosolic Pattern Recognition Receptors	3.53 × 10^−2^	PPIB ↑
Nicotine Degradation II	3.64 × 10^−2^	AOX1 ↑

**Table 2:** Top 5 upstream regulators for each category described in this study, along with their respective *p*-values and target molecules (genes and complexes). Here, “*p*-value of overlap” indicates the significance of overlap between genes of this dataset and those influenced by the upstream regulator, using Fisher’s exact *t*-test [37]. Arrows indicate the direction of gene expression (increased or decreased) in infected versus uninfected mice, based on the averaged expression values for strains included in each response. Additional information for these regulators and molecules, including descriptions and chromosomal locations, is found in Supplementary Table S1.

Top 5 Upstream Regulators	*p*-Value of Overlap	Target Molecules
**Overall (Infected vs. Sham)**		
NFIA ↑	1.37 × 10^−3^	GABRA6 ↓
miR-122-5p (miRNAs w/seed GGAGUGU)	4.42 × 10^−3^	TBX19 ↓
TAF7L ↓	5.16 × 10^−3^	TBX19 ↓
EP300 ↓	2.39 × 10^−2^	TBX19 ↓
GATA2 ↑	2.69 × 10^−2^	TBX19 ↓
Resistant		
MSH2 ↑	1.30 × 10^−5^	IGHG1 ↑, IGKC ↑
IL21R ↑	7.54 × 10^−5^	IGHG1 ↑, IGKC ↑
CXCL10 ↑	1.77 × 10^−4^	*Ccl6* ↓, IGKC ↑
HSP-990	6.24 × 10^−4^	HLA-A ↑
Raet1d ↑/Raet1e ↑	6.24 × 10^−4^	HLA-A ↑
**Resilient**		
MSH2 ↑	4.33 × 10^−5^	IGHG1 ↑, Ighg2b ↑, IGKC ↑
PNPT1 ↑	5.38 × 10^−5^	*Apol9a* ↑/*Apol9b* ↑, GBP6 ↑, IFI16 ↑, IFIH1 ↑, *Oas1b* ↑
Irgm1 ↑	1.54 × 10^−4^	*Apol9a* ↑/*Apol9b* ↑, GBP6 ↑, IFI16 ↑, IFIH1 ↑, *Oas1b* ↑, *Oas1d*↓ (includes others)
IFNB1 ↑	1.63 × 10^−4^	GBP3 ↑, GBP6 ↑, GLP2R ↑, HLA-A ↑, IFI16 ↑, IFIH1 ↑, MCM10 ↑, *Oas1b* ↑, *Oas1d* ↓ (includes others), TRIM6-TRIM34 ↑
ELAVL1 ↓	3.09 × 10^−4^	CASP9 ↓, GBP6 ↑, HLA-A ↑, IFI16 ↑, IFIH1 ↑, *Igha* ↑, *Igkv8-30* ↑, *Oas1b* ↑
**Susceptible**		
GNAS ↑	5.07 × 10^−4^	GDF9 ↓, PRL ↓
BIM 23A760	5.82 × 10^−4^	PRL ↓
IQUB ↑	5.82 × 10^−4^	PRL ↓
RHOQ ↓	5.82 × 10^−4^	PRL ↓
UBE2Q1 ↑	5.82 × 10^−4^	PRL ↓

**Table 3:** Unique biomarkers representing 3 distinct TMEV response profiles were identified from expression data of strains in each category.

Symbol	Entrez Gene Name	Expr Log Ratio	Expr *p*-Value
**Overall**			
STAU1	staufen double-stranded RNA binding protein 1	−0.002	0.999
**Resistant**			
HLA-A	major histocompatibility complex, class I, A	10.265	1.92 × 10^−2^
**Resilient**			
CDPF1	cysteine rich DPF motif domain-containing 1	−1.384	1.62 × 10^−3^
FGF4	fibroblast growth factor 4	−16.685	3.07 × 10^−4^
**Susceptible**			
EIF3J	eukaryotic translation initiation factor 3 subunit J	−6.53	8.03 × 10^−3^
GDF9	growth differentiation factor 9	−3.6	3.57 × 10^−2^
*Gm5148*/*Rps23rg1*	ribosomal protein S23, retrogene 1	−8.529	4.00 × 10^−2^
MID1	midline 1	−5.28	8.03 × 10^−3^
PRL	Prolactin	−17.432	6.52 × 10^−3^

**Table 4:** Numbers of mice evaluated for each of 19 CC strains are shown, separated by sex and infection status. Average levels of TMEV RNA detected in infected mice at 90 dpi, compared to sham-infected mice of the same strain, are reported in the column “TMEV 90 dpi.” We considered negative values to indicate undetectable levels of TMEV RNA. The average 90 dpi cumulative scores for infected mice of each strain (as previously reported, [23]) are listed in the far-right column. Note that phenotypes of sham-infected mice were also evaluated, and used as baseline when scoring infected mice of the same sex and strain.

Strain	Infected F	Infected M	Sham F	Sham M	Total *n*	TMEV 90 dpi	90 dpi Cumulative Score
**CC002**	1	2	1	1	5	−1.52	1.08
**CC005**	2	4	3	3	12	21.90	2.18
**CC006**	3	2	4	2	11	23.98	0.60
**CC011**	3	4	3	3	13	22.55	1.68
**CC015**	1	2	1	2	6	32.38	0.45
**CC017**	1	3	2	3	9	27.56	1.34
**CC023**	1	1	3	2	7	20.95	3.61
**CC024**	1	1	1	0	3	0.15	0.95
**CC025**	1	1	1	0	3	−1.24	1.21
**CC027**	3	1	2	3	9	24.72	0.27
**CC032×CC013**	2	4	1	1	8	−3.60	0.61
**CC036**	1	6	1	2	10	−11.25	0.69
**CC037**	3	4	2	3	12	21.53	0.61
**CC041**	2	2	2	0	6	0.29	1.34
**CC041×CC012**	5	4	2	1	12	20.85	1.35
**CC043**	0	2	1	1	4	20.79	0.84
**CC051**	5	1	2	1	9	−2.67	0.28
**CC058**	1	0	1	1	3	4.48	1.81
**CC072**	0	2	1	0	3	4.60	2.08
**Total**	36	46	34	29	145		

## Data Availability

The data presented in this article are available in Supplementary Materials.

## Supplementary Materials

The following are available online at https://www.mdpi.com/article/10.3390/ijms222111379/s1.
